# Single cell sequencing reveals gene expression signatures associated with bone marrow stromal cell subpopulations and time in culture

**DOI:** 10.1186/s12967-018-1766-2

**Published:** 2019-01-11

**Authors:** Shutong Liu, David F. Stroncek, Yingdong Zhao, Victoria Chen, Rongye Shi, Jinguo Chen, Jiaqiang Ren, Hui Liu, Hee Joon Bae, Steven L. Highfill, Ping Jin

**Affiliations:** 1 Cell Processing Section, Department of Transfusion Medicine, Clinical Center, National Institutes of Health (NIH), 10 Center Drive-MSC-1184, Building 10, Room 3C720, Bethesda, MD 20892-1184 USA; 2 Biometric Research Program, Division of Cancer Treatment and Diagnosis, National Cancer Institute, NIH, Bethesda, MD USA; 3 Center for Human Immunology, Autoimmunity and Inflammation, National Institute of Allergy and Infectious Diseases, NIH, Bethesda, MD USA

**Keywords:** Bone marrow stromal cells, Next generation sequencing, Single cell next generation sequencing, FGFR2, PLAT

## Abstract

**Background:**

Bone marrow stromal cells (BMSCs) are a heterogeneous population that participates in wound healing, immune modulation and tissue regeneration. Next generation sequencing was used to analyze transcripts from single BMSCs in order to better characterize BMSC subpopulations.

**Methods:**

Cryopreserved passage 2 BMSCs from one healthy subject were cultured through passage 10. The transcriptomes of bulk BMSCs from designated passages were analyzed with microarrays and RNA sequencing (RNA-Seq). For some passages, single BMSCs were separated using microfluidics and their transcriptomes were analyzed by RNA-Seq.

**Results:**

Transcriptome analysis by microarray and RNA-Seq of unseparated BMSCs from passages 2, 4, 6, 8, 9 and 10 yielded similar results; both data sets grouped passages 4 and 6 and passages 9 and 10 together and genes differentially expressed among these early and late passage BMSCs were similar. 3D Diffusion map visualization of single BMSCs from passages 3, 4, 6, 8 and 9 clustered passages 3 and 9 into two distinct groups, but there was considerable overlap for passages 4, 6 and 8 cells. Markers for early passage, FGFR2, and late passage BMSCs, PLAT, were able to identify three subpopulations within passage 3 BMSCs; one that expressed high levels of FGFR2 and low levels of PLAT; one that expressed low levels of FGFR2 and high levels of PLAT and one that expressed intermediate levels of FGFR2 and low levels of PLAT.

**Conclusions:**

Single BMSCs can be separated by microfluidics and their transcriptome analyzed by next generation sequencing. Single cell analysis of early passage BMSCs identified a subpopulation of cells expressing high levels of FGFR2 that might include skeletal stem cells.

**Electronic supplementary material:**

The online version of this article (10.1186/s12967-018-1766-2) contains supplementary material, which is available to authorized users.

## Background

Human bone marrow stromal cells (BMSCs), also known as bone marrow-derived mesenchymal stem cells (MSCs), are multipotent cells that have a central role in tissue regeneration, wound healing and maintenance of tissue homeostasis [1–3]. They are involved in a variety of processes such as immunomodulation, hematopoiesis and bone formation. Bone marrow stromal cells have been identified as a promising cell therapy for left ventricular failure due to ischemic heart disease, neurological disorders such as ischemic stroke and many other conditions [4–7].

BMSCs are heterogeneous and highly plastic; their phenotype is dependent on the state of their microenvironment [8]. BMSCs have at least two subpopulations: a skeletal stem cell population and a stromal cell population. Skeletal stem cells differentiate into bone, cartilage and fat. Stromal cells modulate immune function and inflammation, are involved in wound healing, and promote angiogenesis [9]. While BMSCs are being used in many clinical trials, the results have varied. This may be partially due to differences in BMSC manufacturing methods or the numbers of passages used to produce the final BMSC products. Our previous study showed some changes in BMSCs that were associated with time in culture, and we found that stem cell related genes, including Wnt and Notch signaling genes, were down-regulated in late passage BMSCs, suggesting that the early, middle and late passages of BMSCs may have different subpopulation ratios and different functions [10]. Limited by detection technologies, the characteristics of BMSC subpopulations are not completely understood. However, technology is now available for the evaluation of single cells, which allows for the identification and characterization of subpopulations of cells.

Microarray technology is a classic tool used to analyze gene expression profiling, but its usefulness is limited by the need for pre-selected probes of known transcripts and by results based on the analysis of mixed subpopulations of cells. On the other hand, RNA sequencing (RNA-Seq) offers many advantages for studying BMSCs including the ability to identify novel transcripts and increased sensitivity and specificity, which may reveal weakly expressed genes previously missed by microarray analysis. Furthermore, single cell RNA-Seq is able to analyze gene expression at the individual cell level, which is helpful for cell-to-cell genetic comparison and the potential identification of cell subpopulations.

In this study, we evaluated several passages of BMSCs from a single subject using both gene expression microarray and RNA-Seq technology. Both the unseparated or bulk cells and the single separated cells were analyzed in order to better understand BMSC subpopulations and changes in subpopulations with BMSC passage.

## Materials and methods

### Cell isolation and culture

Bone marrow collection and BMSC isolation and culture were performed according to a Standard Operating Procedure (SOP) established in our lab as previously described [10]. One vial of frozen passage 2 BMSCs was thawed and plated in T75 flasks following the SOP. During the serial culture, cells were seeded at 5000 cells/cm^2^ and harvested at 70–80% confluence. From passages 3 to 8, cells were passaged after 5 days of culture, and culture media was changed on day 3; from passages 9 to 10, the cells were passaged after 8 days of culture, and culture media was changed on day 3 and 6. Cells from each passage were cryopreserved in a solution containing 90% FBS and 10% DMSO for further studies.

### Flow cytometry analysis

BMSC surface markers were analyzed by flow cytometry. The cells were stained with: CD73 (CD73-PE, BD Bioscience, San Diego, CA), CD105 (CD105-APC, eBioscience, San Diego, CA), CD146 (CD146-PE, BD Bioscience), CD44 (CD44-APC, BD Bioscience), and isotype control. Data were collected on a FACSCalibur (BD Bioscience) and analyzed using FlowJo software (Tree Star, Inc., Ashland, OR). Greater than or equal to 80% of the cells expressed CD105, CD73 and CD90, and ≥ 60% of the cells expressed CD146, as measured by flow cytometry, and ≤ 5% of the cells expressed CD45, CD14, CD34, and CD11b.

### RNA isolation and quality control

Total RNA extractions were performed on samples from passages 2, 3, 4, 6, 8, 9, and 10 using RNeasy Mini Kit (Qiagen) according to the manufacturer’s protocol. RNA was quantified using Nanodrop 8000 (Thermo Scientific, Wilmington, DE). Total RNA quality was evaluated following isolation using a 2100 Bioanalyzer (Agilent Technologies, Santa Clara, CA). Samples with an RNA Integrity Number (RIN) value ≥ 8 were used for bulk cell RNA-Seq and MicroArray for gene expression analysis.

### Gene expression analysis of unseparated cells using microarrays

Microarray gene expression analysis was performed on 4 × 44 K Whole Human Genome Microarrays (Agilent Technologies, Santa Clara, CA, USA) according to the manufacturer’s protocol. In general, 200 ng of total RNA from each sample was amplified, labeled, and hybridized on the array chip using a Quick Amp Labeling kit (Agilent). Array images were obtained by Agilent Scan G2600D. Then images were extracted using Feature Extraction 12.0 software (Agilent). Partek Genomic Suite 6.4 (Partek Inc., St. Louis, MO, USA) was used for data visualization and hierarchical cluster analysis.

### Bulk cell mRNA next generation sequencing

cDNA library preparation was performed using the TruSeq Stranded Total RNA Library Prep Kit (Illumina, USA) according to the manufacturer’s protocol. In brief, the polyAcontaining mRNA molecules were purified from 4 μg of total RNA for each sample. After library preparation, cDNA library templates were generated. 2100 Agilent High Sensitive DNA chips (Agilent) were used for quality control. KAPA Library Quantification Kit (BioRad) was used for quantification and normalization before loading the samples onto an Illumina NextSeq 500 instrument for sequencing. Nextera 500 High Output v2 kit (150 cycles) was used for bulk cell RNA sequencing (RNA-Seq). PhiX Control v3 (Illumina) was used as a quality control for sequencing runs.

### Single cell mRNA next generation sequencing

Frozen cells from different passages were thawed and plated in T75 flasks for 5–7 days. Once they reached 70–80% confluence, cells were collected for single cell separation. Fluidigm C1 Single-Cell Auto Prep Array for mRNA-Seq (10–17 μm) and Fluidigm C1 system were used for cell capture following the Fluidigm protocol, then reverse transcription and cDNA amplification were performed using the SMARTer PCR cDNA Synthesis kit (Clontech, version 1) on the C1 unit.

Single-cell cDNA from validation cells were harvested for library preparation. Nextera XT DNA sample preparation kits and Index kits were used for library preparation following the manufacturer’s protocol. Single-cell cDNA libraries from each passage were pooled and cleaned up, then cDNA size distribution was examined by 2100 Bioanalyzer. This was followed by quantification and normalization with the KAPA Library Quantification Kit and BioRad CFX96 qPCR machine (BioRad). Single cell mRNA sequencing was performed on NextSeq 500 by using the NextSeq High Output v2 kit (300 cycles). PhiX Control v3 (Illumina) was used as a quality control for sequencing runs.

### Sequencing data analysis

BCL files generated by Illumina NextSeq were converted to standard FASTQ files following Illumina’s protocol (basespace.illumina.com). FASTQ files were imported to Partek Flow (Partek Inc., St. Louis, MO, USA) for base trimming, alignment (STAR 2.4.1d, hg19-RefSeq Transcripts 2016-5-2), and quality control. Reads were then quantified to annotation model (Partek E/M, hg19-RefSeq Transcripts 2016-5-2) to generate raw gene counts.

For bulk cell next generation sequencing, DESeq2 [11] was used for differential expression and pcaExplorer (https://github.com/federicomarini/pcaExplorer) for data visualization. For single cell next generation sequencing, SC3 [12] (http://biorxiv.org/content/early/2016/09/02/036558), scater [13] destiny were used for data visualization. Ingenuity Pathway Analysis (IPA) was used for functional pathway analysis.

## Results

### Gene expression analysis of unseparated BMSCs by microarray and RNA-Seq

#### Principal component analysis of microarray and RNA-Seq gene expression data

**Fig. 1:**
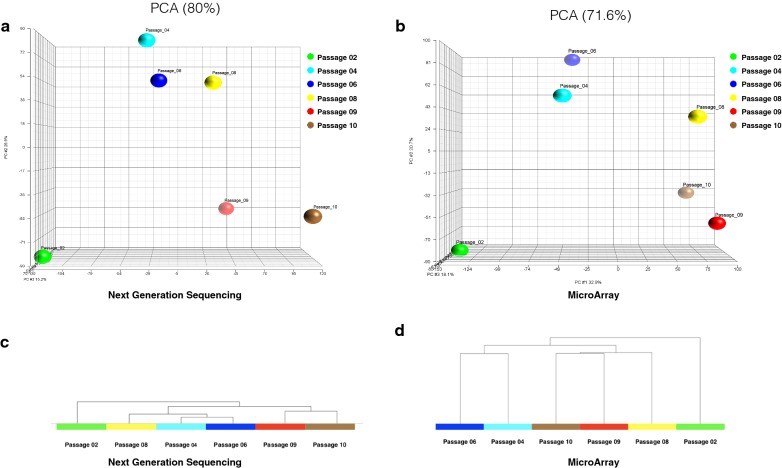
Transcriptome analysis of serial passages of unseparated BMSCs using microarrays and next generation sequencing. PCA analysis of gene expression data from passages 2, 4, 6, 8, 9 and 10 BMSCs obtained by RNA-Seq are shown in **a** and by microarray analysis in **b**. Similarity matrix analysis of BMSC transcriptomes obtained by RNA sequencing is shown in **c**. Hierarchical clustering analysis of BMSC transcriptomes obtained using gene expression microarrays is shown in **d**

To validate RNA-Seq technology, we evaluated unseparated or bulk BMSCs by RNA-Seq and traditional microRNA gene expression analysis. Six BMSC passages (passages 2, 4, 6, 8, 9 and 10) were analyzed. Principle Component Analysis (PCA) based on the entire RNA-Seq (Fig. 1a) and microarray data sets (Fig. 1b) showed similar results. Passages 4 and 6 cells were grouped together as were passages 9 and 10 cells, but the two groups were distinct from each other. Similarity matrix analysis of RNA-Seq data (Fig. 1c) and hierarchical clustering analysis of the microarray data also grouped passages 4 and 6 together as well as passages 9 and 10 (Fig. 1d). RNA-Seq found that passage 8 cells were similar to passages 4 and 6 cells while microarray analysis found that passage 8 cells were separate from passages 4 and 6 group and passages 9 and 10 group. These data highlight the consistency of microarray and RNA-Seq technology when used to analyze BMSCs in bulk.

#### Expression of classic BMSC makers as determined by RNA-Seq analysis of unseparated cells

**Fig. 2:**
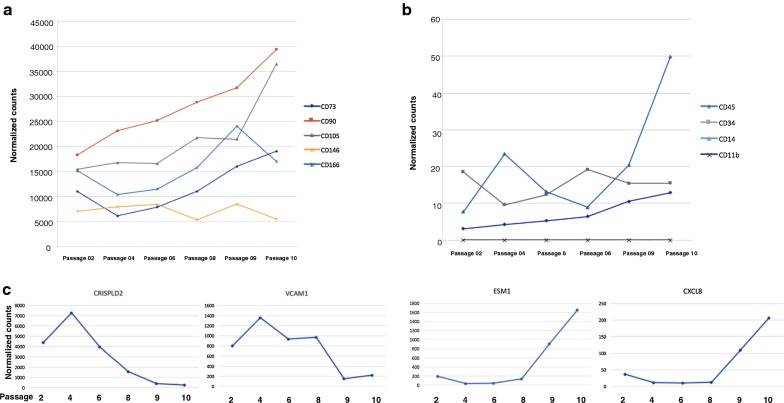
Expression of stromal cell marker genes by BMSCs as measured by RNA-Sequencing. The transcriptome of BMSCs from passages 2, 4, 6, 9 and 10 was analyzed by RNA-Seq. The expression of stromal cell markers CD73, CD90, CD105, CD146 and CD166 are shown in **a** and the expression of hematopoietic cell markers in CD45, CD34, CD14 and CD11b in **b**. The expression of four genes differentially expressed among early and late passage cells is shown in **c**. Among the genes whose expression was greatest in early passage BMSC, the fold difference was greatest for CRISPLD2, and VCAM1 is a functionally important gene. Among the genes whose expression was greatest in late passage BMSCs, the fold difference was greatest for ESM1, and CXCL8 is a functionally important gene

According to the ISSCR (International Society for Stem Cell Research) and ISCT (International Society for Cellular Therapy) [14], MSCs should express CD90 (THY1), CD73 (NT5E), CD105 (ENG), CD146 (MCAM) and CD166 (ALCAM), but lack expressions of CD11b, CD14, CD45 and CD34. After normalizing the bulk BMSC RNA-Seq data, the expression of these markers was analyzed (Fig. 2a, b). The RNA-Seq gene expression analysis showed that all passages expressed the classic surface markers CD73, CD90 and CD105 and that their expression increased with each passage. All BMSC passages also expressed CD146 and CD166. The expression of CD166 increased with each passage through passage 9, but the expression of CD146 changed very little (Fig. 2a). As expected, the expression of CD14, CD34 and CD45 was minimal (Fig. 2b). CD11b was not expressed by any of the passages. The expression of CD14 increased slightly with passages (Fig. 2b).

#### Identification of differentially expressed genes among early and late passage unseparated BMSCs using RNA-Seq

Based on the unsupervised clustering analysis of both the RNA-Seq and microarray data, we classified passages 4 and 6 cells as early passages and passages 9 and 10 as late passages. Then we compared genes differentially expressed in early and late BMSC passages. We identified a set of 532 differently expressed genes using the RNA-Seq data (adjusted p value < 0.01, log2 fold change > 1) (Additional file 1: Table S1). The 25 genes with the greatest fold decrease among early passage BMSCs compared to late passage BMSCs and the 25 with the greatest fold increase in early passage BMSCs compared to late passage cells are shown in Table 1.

**Table 1:** The 25 genes with the greatest fold decrease and 25 genes with the greatest fold increase among early passage BMSCs compared to late passage

25 genes with greatest fold decrease			25 genes with greatest fold increase		
Gene name	Fold change (Log2)	adj.p	Gene name	Fold change (Log2)	adj.p
CRISPLD2	3.438522292	2.15E−31	ESM1	− 4.06046951	1.62E−40
BZRAP1	3.435446804	3.23E−30	LMO2	− 3.521932491	1.95E−27
HR	3.358320968	1.07E−24	MYCT1	− 3.224349082	2.39E−22
DDIT4L	2.878150028	1.37E−22	ITGA2	− 3.130634867	2.28E−71
COLEC12	2.867107804	5.03E−37	FAM180A	− 3.071039961	8.57E−25
KRT23	2.734415717	5.29E−17	LINC01468	− 3.050530756	5.72E−14
TSPAN18	2.3851619	2.04E−13	PLAT	− 3.02360288	2.68E−21
CCKAR	2.311341723	1.30E−09	PCDH10	− 2.827869011	1.11E−22
ITGB8	2.303574161	9.65E−34	DHRS9	− 2.765618004	4.80E−11
OLFML2B	2.292413715	8.62E−14	SHANK2	− 2.613657452	2.81E−20
VCAM1	2.2814846	2.75E−16	BMP6	− 2.611030064	1.78E−15
VWA1	2.276678575	2.50E−09	DUSP4	− 2.574836081	1.65E−12
INHBE	2.259102004	2.37E−11	MYPN	− 2.565054834	8.10E−13
RBP1	2.210166017	1.17E−08	OLR1	− 2.528322633	2.36E−15
PIM1	2.141091315	2.35E−15	PRSS3	− 2.526358137	2.35E−09
FGFR2	2.104662461	6.31E−12	CXCL8	− 2.525510167	2.50E−10
DCLK1	2.10464492	1.54E−16	IL13RA2	− 2.509289508	5.46E−09
BAALC	2.081623838	1.16E−07	P2RX5	− 2.383293998	3.68E−11
RCOR2	2.021028143	1.99E−06	PCOLCE2	− 2.343508238	4.83E−16
DES	1.973048748	9.27E−06	HIST1H1C	− 2.335473076	4.01E−18
LRRC2	1.960590792	1.37E−08	KCNC4	− 2.284954823	2.29E−22
FBXL22	1.940950509	5.91E−06	COL13A1	− 2.224222192	4.53E−13
ABCC3	1.935121057	1.26E−10	LIPG	− 2.17991959	1.85E−08
NREP	1.921436126	6.94E−16	C11orf87	− 2.161091526	3.82E−10
ARHGAP28	1.890479851	4.54E−08	CALB2	− 2.147443644	3.62E−09

Next, we selected the genes Cysteine-rich secretory protein LCCL domain-containing 2 (CRISPLD2) and Endothelial cell-specific molecule 1 (ESM1), which had the largest fold changes in early and late passage cells respectively (Table 1) and investigated their expression in all 6 passages. CRISPLD2 expression was high in early passage cells and then decreased gradually beyond passage 4 and ESM1 expression increased gradually from passage 4 (Fig. 2c). We also investigated the expression of Vascular Adhesion Molecule 1 (VCAM1) and CXCL8, which were among the most differentially expressed genes and which have been shown to be important BMSC functional genes. The expression of VCAM1 was greatest on passage 4 cells and then decreased with passage while that of CXCL8 increased after passage 8 (Fig. 2c).

**Fig. 3:**
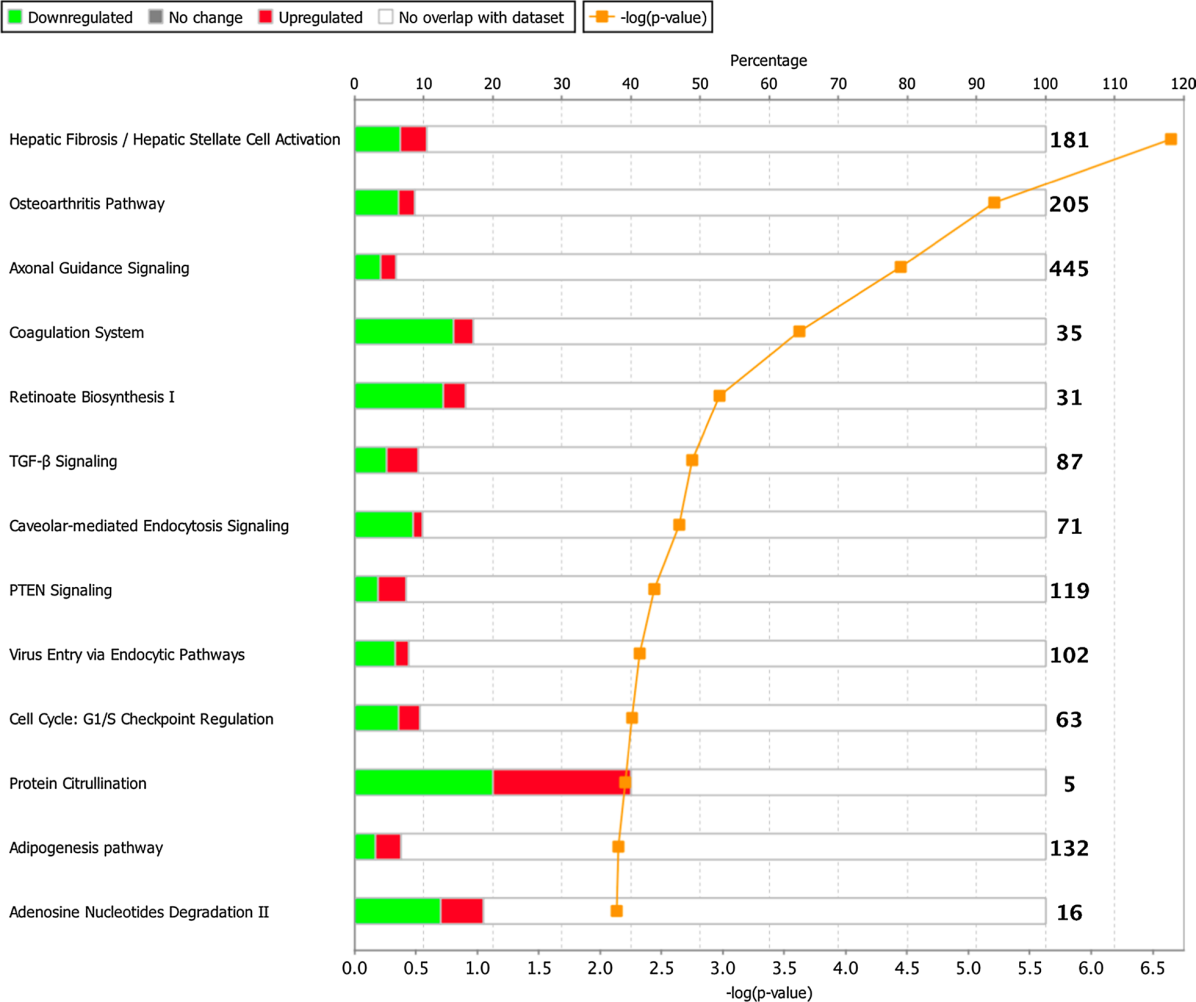
Ingenuity Pathway Analysis (IPA) of 532 genes differently expressed among early and late passage BMSCs. Unseparated BMSCs were analyzed by RNA sequencing. The transcriptome of early passage BMSCs, passages 4 and 6, was compared with the transcriptome of late passage BMSCs, passages 9 and 10, and 523 genes were found to be differentially expressed (adjusted p value < 0.01, log2 fold change > 1). The 532 genes were subjected to IPA analysis

The 532 differently expressed genes were analyzed by Ingenuity Pathway Analysis (IPA) and they were found to be present in a variety of pathways (Fig. 3). We also compared the 532 genes identified by RNA-Seq to the 155 genes recognized in our previous study as being highly correlated with BMSC age or time in culture as determined by microarray gene expression analysis [10]. From this comparison, we identified 49 genes present in both data sets (Additional file 2: Table S2). Among these, PLAT had the largest fold change in early passage BMSCs in the current RNA-Seq data set. The 49 genes also included Runt Related Transcription Factor 2 (RUNX2), a gene associated with bone marrow stromal cell osteogenesis and a key gene in the Fibroblast Growth Factor (FGF) pathway.

### Analysis of single BMSCs using RNA-Seq

#### Single cell transcriptome analysis reveals BMSC subpopulations change with passage

The analysis of unseparated BMSCs showed that RNA-Seq and microarray gene expression analysis yielded consistent results and led us to analyze BMSCs with single cell mRNA next generation sequencing. We performed single cell RNA-Seq analysis on five selected BMSC passages. Analysis of unseparated BMSCs revealed that passage 2 cells were outliers in both RNA-Seq and microarray platforms. This may be because the passage 2 cells were thawed and cultured for 48 h. We therefore used passage 3 instead of passage 2 for single cell RNA-Seq analysis. Upon harvesting passage 10 BMSCs, we also found that they had various cell morphologies and sizes, which resulted in low single cell capture efficiency. Thus, they were also excluded from this analysis.

**Fig. 4:**
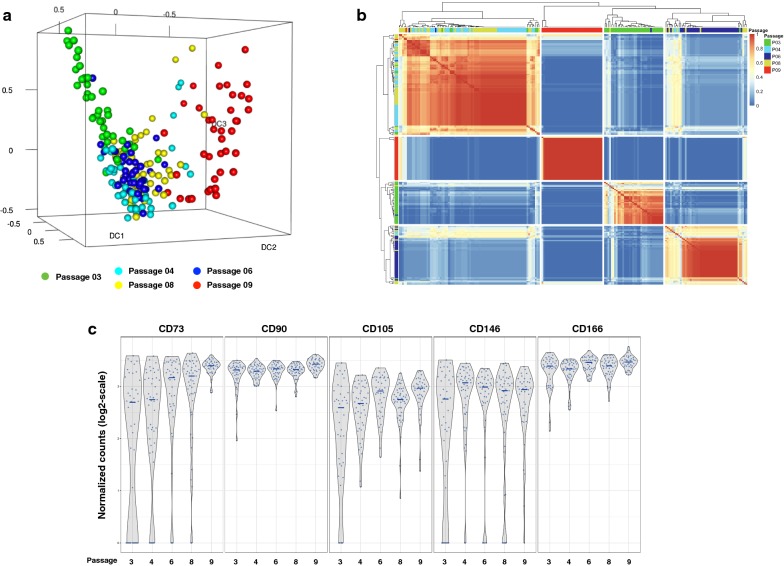
Single cell transcriptome analysis of passages 3, 4, 6, 8 and 9 BMSCs. BMSCs from each passage were separated using a microfluidics platform and were analyzed using RNA-Sequencing. The results were analyzed using 3D Diffusion map visualization (**a**) and similarity matrix (**b**). The expression of stromal cell markers CD73, CD90, CD105, CD146 and CD166 on single cells are shown in **c**. Passage 3 cells are shown in green, passage 4 cells in light blue, passage 6 cells in dark blue, passage 8 cells in yellow, and passage 9 cells in red

We first evaluated the overall distribution of all passages analyzed by single cell whole transcriptome RNA-Seq using a 3D Diffusion map visualization of 209 individual cells, color-coded by passage number (Fig. 4a). This analysis showed spatial overlap or clustering of passage 4, passage 6 and passage 8 cells. Passages 3 and 9 were mostly isolated from the other passages. For passage 3 cells, some were uniquely separate from all others while some overlapped with passages 4 and 6 cells. All of passage 9 cells were separate from the other cells. These results are comparable to those obtained by the analysis of unseparated BMSCs.

The single cell RNA-Seq data were analyzed using 209 × 209 consensus matrix assessing similarity of cells through cluster analysis (Fig. 4b). The 209 columns and rows represent the 209 individual cells that were analyzed in pair-wise comparisons for similarity. Similarity was scored from 0 (blue) to 1 (red) after analyzing clustering results from all potential parameters, where 0 means the pair in question is never in the same cluster and 1 means they are always in the same cluster. The matrix was also organized by hierarchical clustering. Most cells clustered according to their passage number. A few cells were grouped with cells from other passages. A small assorted group was also present with cells from many different passages.

Overall, there were two major clusters; one cluster of primarily passages 4 and 8 cells and a second cluster of passages 3, 6 and 9 cells. Within the latter, there were three subgroups; one subgroup of mostly passage 9 cells, one subgroup of mostly passage 3 cells and one subgroup of mostly passage 6 cells. Since these results differed somewhat from our unseparated cell results, we elected to further evaluate the BMSC subpopulations by assessing expression of specific markers in single BMSCs across passages.

#### Expression of BMSC markers by single cells

The expression of classic BMSC markers evaluated on unseparated BMSCs were also investigated at the single cell level among five passages (Fig. 4c). The expression of positive markers (CD73, CD90, CD105, CD146 and CD166) as determined by single cell RNA-Seq showed a similar trend as the unseparated cell analysis (Fig. 2a), but a few early passage cells did not express CD73, CD105 and CD146. Single cell analysis found that the expression of CD45, CD34 and CD14 was very low and CD11b was not expressed by any of the cells tested (data not shown). This indicated that analysis of BMSCs by single cell RNA-Seq and unseparated cell RNA-Seq yielded similar results.

#### Identification of genes predictive of BMSC passage number

**Fig. 5:**
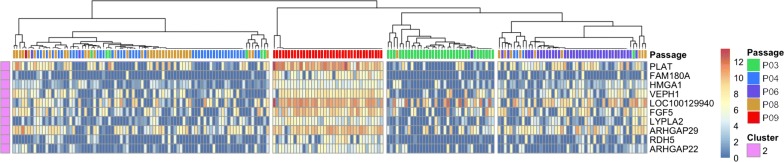
Hierarchical clustering analysis of the expression of 10 genes predictive of single BMSC time in culture. The binary classification algorithm, based on the mean gene expression for each cluster, was used for gene expression rankings to generate marker predictions which were assessed with receiver operating characteristic (ROC) curves and Wilcoxon signed rank test for p-values. The resulting 10 genes that meet marker gene definitions of area under the ROC curve (AUROC) > 0.85 and p-value < 0.01 are shown. The expressions of these 10 genes among single BMSCs from passages 3, 4, 6, 8 and 9 were analyzed by hierarchical clustering analysis

Next, we used the single cell RNA-Seq data to identify genes that predicted BMSC time in culture or passage number. The binary classification algorithm, based on the mean gene expression for each cluster, was used for gene expression rankings to generate marker predictions. Predictions were assessed with receiver operating characteristic (ROC) curves and Wilcoxon signed rank test for p-values. The resulting genes that meet marker gene definitions of area under the ROC curve (AUROC) > 0.85 and p-value < 0.01 are shown in Fig. 5. The matrix also shows the log-transformed expression values for each gene. 10 passage-associated genes were identified.

#### Markers of late BMSC passages

**Fig. 6:**
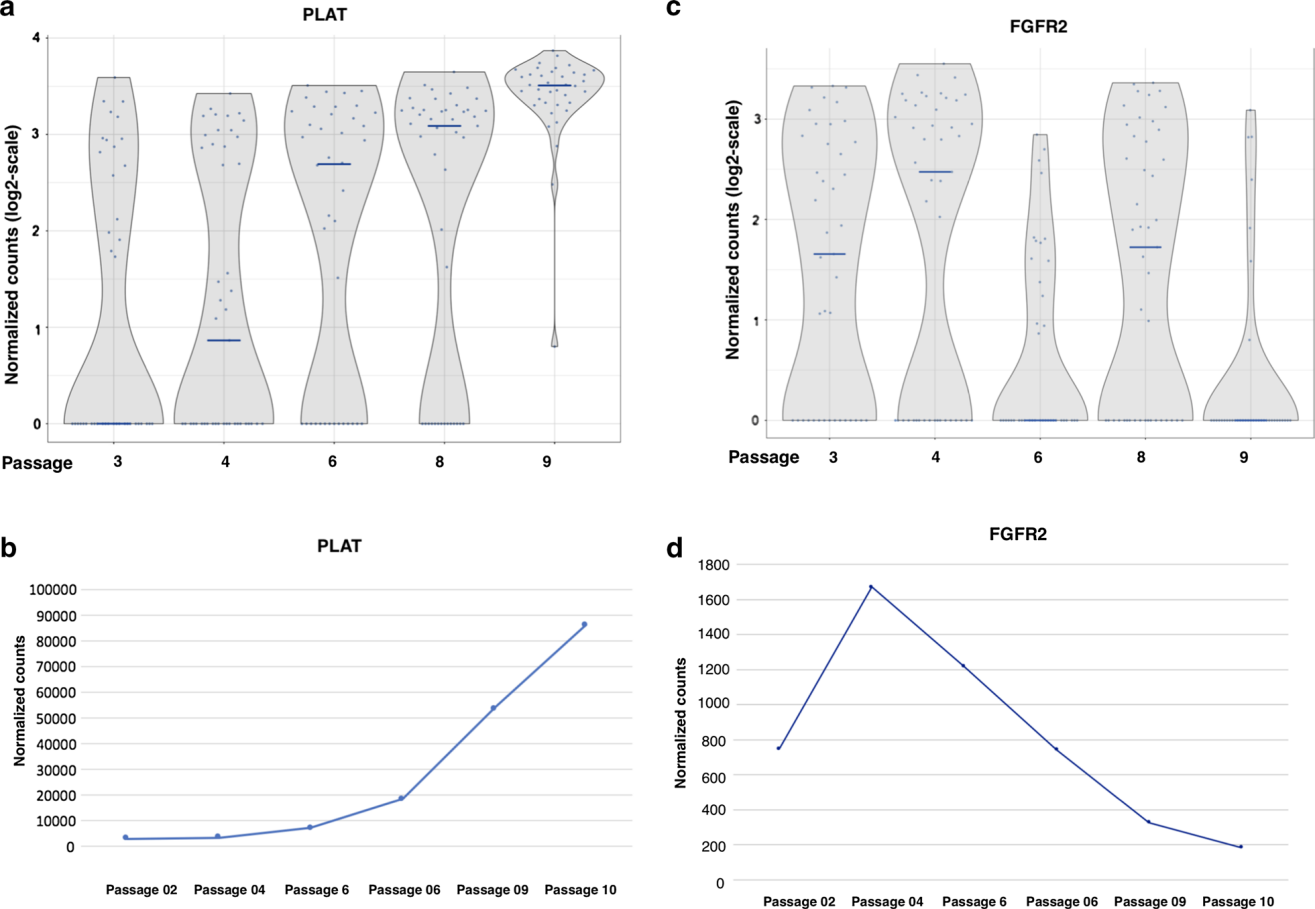
The expression of PLAT and FGFR2 by single BMSCs RNA-seq from passages 3, 4, 6, 8 and 9. The expression of a gene more highly expressed in late passage BMSCs, PLAT, was assessed by single cell RNA-Sequencing (**a**) and RNA sequencing of unseparated BMSCs (**b**). The expression of FGFR2, which is more highly expressed in early passage BMSCs, was assessed by single cell RNA sequencing (**c**) and sequencing of unseparated BMSCs (**d**). Single cell PLAT expression by each cell was plotted as individual data points and the mean expression level for each passage is shown by a horizontal line

We reviewed the expression of all 10 passage predictive genes. For all 10 genes, the expression of each of the genes by single cells from early passages was less than the expression by single cells from late passages (Additional file 3: Figure S1). Among the 10 passage predictive genes, the expression of Plasminogen Activator, Tissue Type (PLAT), was the most elevated in passage 9 cells, making it a potential marker for BMSC subpopulations that were most prominent among late passage cells. PLAT expression was assessed across all five passages using the single cell RNA-Seq data (Fig. 6a). As the passage number increased, the mean expression level of PLAT and the number of cells expressing PLAT both increased significantly. Analysis of PLAT expression of unseparated cells by RNA-Seq data showed a similar trend (Fig. 6b). In addition, the RNA-Seq data revealed that PLAT had the largest fold change (Additional file 1: Table S1), demonstrating that it is a key gene associated with a subpopulation more prevalent in late passage BMSCs and that it is a potential marker for BMSC senescence.

#### Markers for early passage BMSCs

To further understand BMSC single cell subpopulations, we hoped to identify markers with high expression specifically in early passage cells. Since the expression of all 10 passage predictive genes was greater among late passage single cells, we reviewed the genes whose expression was greater in early passage cells as determined by RNA-Seq analysis of unseparated BMSCs. Among the genes whose expression was increased most in early passage BMSC, we identified Fibroblast Growth Receptor 2 (FGFR2), VCAM1, Integrin Subunit Beta 8 (ITGB8) (Table 1) and RUNX2 (Additional file 1: Table S1) as potential markers.

Both FGFR2 and RUNX2 are involved in bone formation and belong to the FGF pathway. Coutu et al. showed FGF-2 protects skeletal stem cells from senescence and helps to maintain their stem cell characteristics [15]. Our previous paper revealed that BMSCs cultured with FGF-2 had better bone formation ability [16]. RUNX2 is a well-studied and downstream gene in the FGF pathway. This suggested that FGFR2 and RUNX2 may be important markers of BMSC skeletal stem cells and early passage BMSCs.

**Fig. 7:**
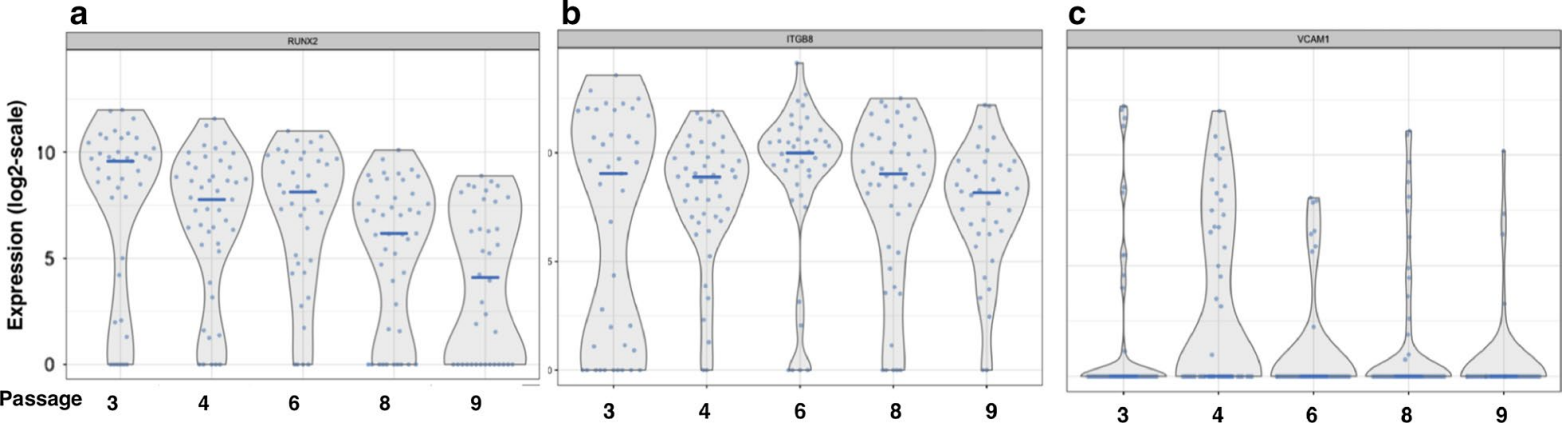
Single cell expression of RUX2, ITGB8 and VCAM1 by BMSCs from passages 3, 4, 6, 8 and 9. The expressions by single BMSCs of three genes whose expression was greater in early passage BMSCs, RUNX2 (**a**), ITGB8 (**b**) and VCAM1 (**c**), are shown

All four markers (FGFR2, RUNX2, VCAM1 and ITGB8) were expressed by some but not all single BMSCs. Evaluation of FGFR2 expression by single BMSCs using RNA-Seq found that its expression among single BMSCs increased from passages 3 to 4, fell from passages 4 to 6 and then increased again in passage 8 before falling to very low levels in passage 9 as determined by single cell RNA-Seq (Fig. 6c) and bulk cell RNA-Seq analysis (Fig. 6d). The proportion of cells expressing RUNX2 and intensity of expression of reactive cells decreased with passage number (Fig. 7a). The proportion of cells expressing ITGB8 was similar for all passages and the intensity of expression of ITGB8 was slightly greater among passage 6 cells compared to the other passages and was least in passage 9 cells (Fig. 7b). For all passages, few cells expressed VCAM1, but passage 4 had the greatest proportion of cells that expressed VCAM1 (Fig. 7c). The results of this single cell analysis show that changes in the expression of surface markers are more complex than apparent by analysis of unseparated cells.

Interestingly, Fibroblast growth factor 5 (FGF-5) showed in Fig. 5 is a secreted heparin-binding growth factor that binds to FGF receptors 1 and 2 (FGFR1 and FGFR2) [17]. The administration of rhFGF5 to tonsil-derived mesenchymal stem cells could increase osteogenic differentiation of Human Tonsil-Derived Mesenchymal Stem Cells [18].

#### BMSC early passage cell subpopulations

**Fig. 8:**
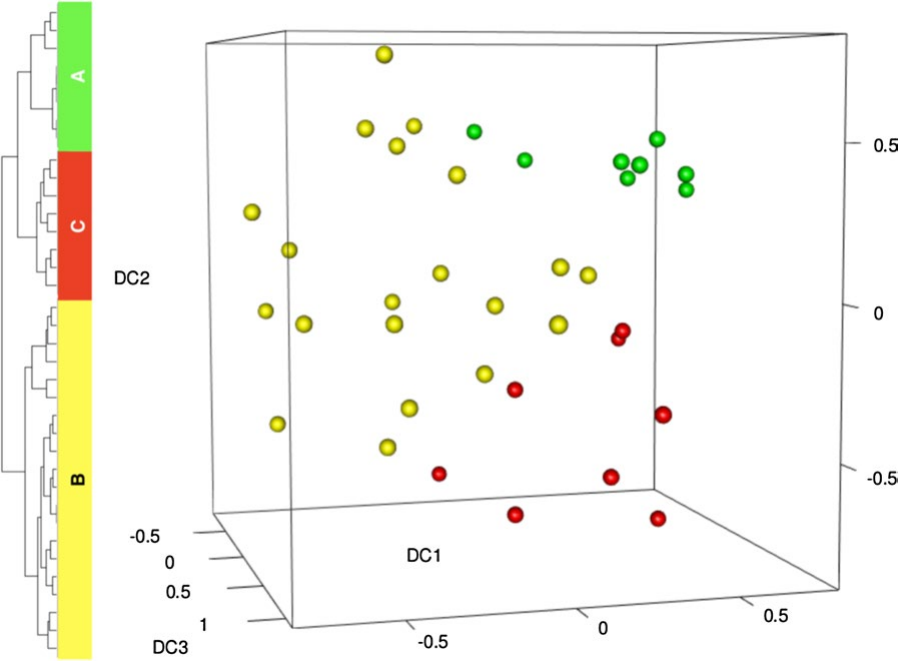
Identification of BMSC subpopulations within passage 3 cells. BMSCs from passage 3 were analyzed by single cell RNA sequencing. The transcriptome of 36 cells was analyzed by 3D Diffusion map visualization, and 3 BMSC subpopulations were identified. Cells in subpopulation A are show in green, subpopulation B in yellow, and subpopulation C in red

**Fig. 9:**
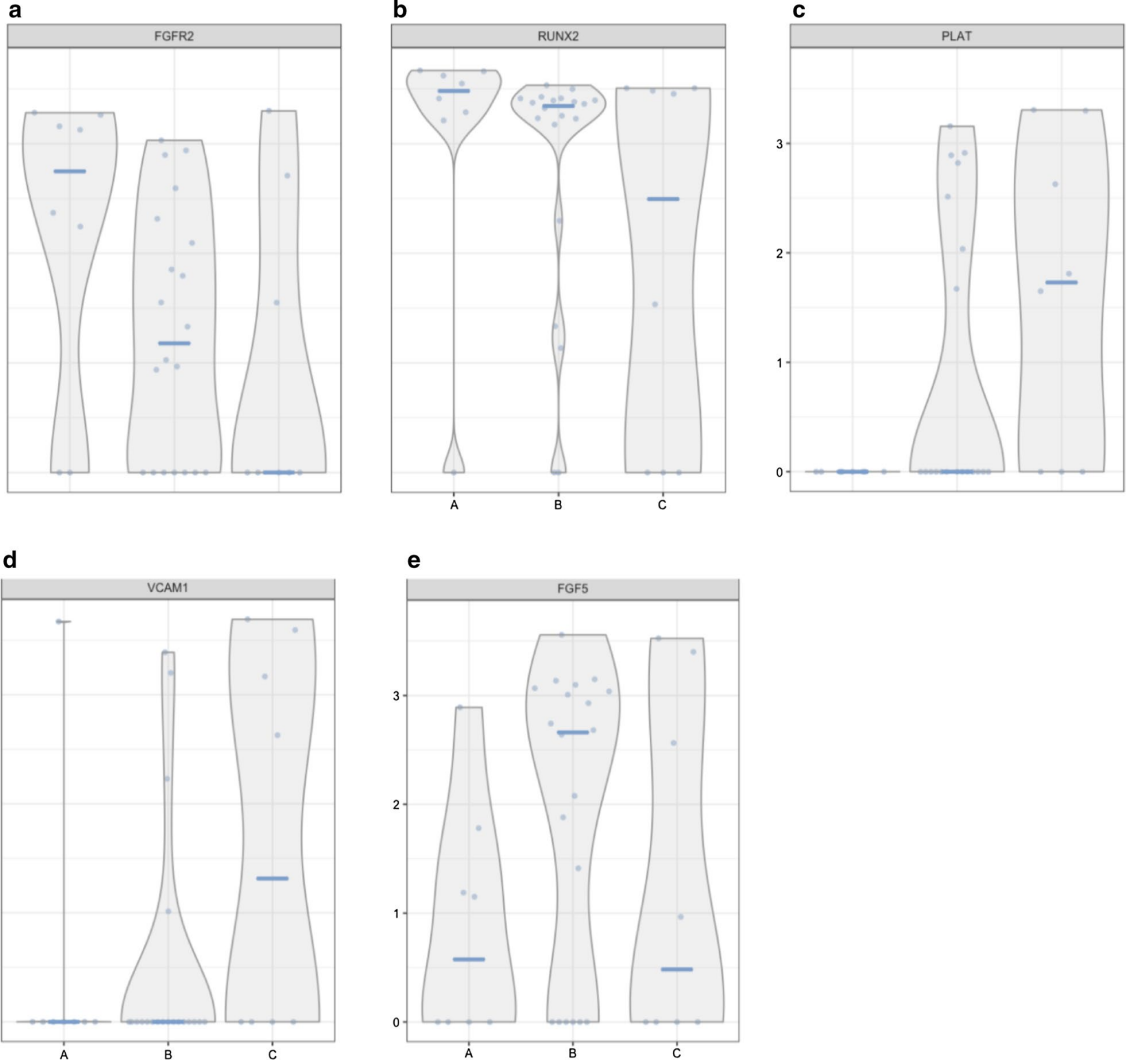
Expression of markers of early and late passage BMSCs by passage 3 subpopulations. The single BMSCs in subpopulations A, B and C of passage 3 BMSCs were analyzed for the expression of early passage BMSC markers, FGFR2 (**a**) and RUNX2 (**b**), and late passage markers, PLAT (**c**) and VCAM1 (**d**). The subpopulations were also analyzed for the expression of the FGFR2 related gene, FGF5 (**e**)

The BMSC skeletal stem cell population is poorly characterized but is lost with serial passage of BMSCs. To better characterize the BMSC skeletal stem cell subpopulation, we analyzed single cell RNA-Seq data from matrix cluster 3 cells which were made up primarily of passage 3 cells with some passage 6 cells and one passage 8 cell (Fig. 4b). 3D Diffusion map visualization of cluster 3 cells based on single cell RNA-Seq data is shown in Fig. 8. Groups of cells were separated into Group A, B, and C (Fig. 8). Subpopulation B was the largest and subpopulations A and C were smaller and of approximately equal size. Next, we investigated the expression of markers of early passage BMSCs, FGFR2, RUNX2, VCAM1 and FGF5, and the later passage marker PLAT among the different groups (Fig. 9). Group A had the highest expression of FGFR2 but the lowest of PLAT. Both FGFR2 and PLAT expression levels in Group B were between that of Groups A and C (Figs. 6d, 9). Group A cells also expressed high levels of RUNX2 and low levels of FGF5 and VCAM1; Group C cells expressed high levels of VCAM1 and RUNX2; and Group B cells expressed high levels of RUNX2, FGF5 and low levels of VCAM1 (Fig. 9 and Table 2).

**Table 2:** Expression of FGFR2, RUNX2, FGF5, VCAM1, PLAT and ITGB8 by single cells in cluster 3

Gene	Cell group		
	A	B	C
FGFR2	4+	2+	0
RUNX2	4+	4+	3+
FGF5	1+	3+	1+
VCAM1	0	0	2+
PLAT	0	0	3+

We also probed passage 9 cells for the presence of these three BMSC subpopulations and discovered that they consist of a mostly uniform population with high PLAT expression and no FGFR2 expression.

## Discussion

This study showed that transcriptomes of single BMSCs can be analyzed by RNA-Seq. We first showed the results of global transcriptome analysis of unseparated BMSCs using RNA-Seq and microarrays yielded similar results. The analysis of serial passages of unseparated BMSCs using RNA-Seq and microarrays resulted in a similar separation of BMSC passages and yielded similar sets of genes that characterized early and late BMSC passages.

The analysis of several BMSC passages from the same healthy subject using single cell RNA-Seq yielded slightly different results from the analysis of unseparated cells. While the analysis of serial passages of unseparated BMSCs by RNA-Seq found distinct differences among each passage, single cell analysis found considerable overlap in the transcriptome of cells from some passages. While cells from very early and very late passages, namely passages 3 and 9, were distinct from other passages, there was considerable overlap among cells from the middle passages, passages 4, 6 and 8.

The RNA-Seq analysis identified FGFR2 as a marker of early passage BMSCs and PLAT as a marker of late passage BMSCs. FGFR2 is one of the receptors in the FGFR family, which includes FGFR1, FGFR2, FGFR3, and FGFR4. The main function of FGFR family is to regulate FGF signaling pathway, and FGF signaling contributes to cell self-renewal, senescence and osteogenesis in stem cells [19]. The higher sensitivity of next generation sequencing likely contributed to discovering the expression change in FGFR2. The single cell RNA-seq analysis found that with increased time in culture, the expression of FGFR2 decreased. While most single BMSCs in all passages expressed the MSC markers CD90 (THY1), CD73 (NT5E), CD105 (ENG), and CD166 (ALCAM), a portion of BMSCs did not express FGFR2, regardless of passage number and among passage 9 BMSCs only a few cells expressed FGFR2. This suggests that FGFR2 is a marker of the skeletal stem cell. This is supported by our previous study which showed that adding FGF-2 during BMSC expansion enhanced in vivo osteogenic capacity [16].

The analysis of the transcriptome of single BMSCs from early passage BMSCs allowed for the identification and partial characterization of three distinct BMSC subpopulations. One subpopulation was characterized by the strong expression of FGFR2 and weak FGF5 and lack of expression of PLAT. Since FGFR2 was more likely to be expressed by early passage cells and PLAT by late passage cells and since FGFR2 is involved in osteogenesis, this subpopulation may be skeletal stem cells. A second and the largest BMSC subpopulation expressed lower levels of FGFR2 but higher levels of FGF5 and low levels of PLAT. This subpopulation may represent differentiating skeletal stem cell progenitors. A third BMSC subpopulation was characterized by strong expression of PLAT and VCAM1, but lower levels of FGF5 and no FGF2. Because VCAM1 promotes angiogenesis, this subpopulation may be involved with angiogenesis.

## Conclusion

This study demonstrated that single cell RNA sequencing technology can be used with BMSCs. We used single cell RNA sequencing to characterize the changes that occur in BMSCs associated with time in culture. The study is limited by the fact that cells from a single healthy subject were studied. Additional studies are needed which include BMSCs from a greater number of subjects. Furthermore, new, more robust platforms for single cell RNA sequencing will allow for better characterization of these BMSC subpopulations. However, the results of this study were consistent enough to identify BMSC subpopulations and partially characterized them.

## Additional files

**Additional file 1: Table S1.** Gene list.

**Additional file 2: Table S2.** Gene list.

**Additional file 3: Figure S1.** Gene expressions in single cells.

## Abbreviations

BMSC: bone marrow stromal cells
PCA: principle component analysis
RNA-Seq: ribonucleic acid sequencing
