# Supplementary material for: Single cell sequencing reveals gene expression signatures associated with bone marrow stromal cell subpopulations and time in culture
Source: J Transl Med. 2019 Jan 11;17:23. doi: 10.1186/s12967-018-1766-2 (PMC6330466; doi:10.1186/s12967-018-1766-2)
Supplement: Supplementary file 2 — Additional file 2: Table S2. Gene list. [file 12967_2018_1766_MOESM2_ESM.docx]

**Additional File 2 Table S2**: 49 genes common between data set of genes highly correlated with BMSC age and time in culture and data set of 532 differentially expressed genes when comparing early vs late BMSC passages.

|  | baseMean | log2FoldChange | lfcSE | stat | pvalue | padj |
| --- | --- | --- | --- | --- | --- | --- |
| DDIT4L | 501.102 | 2.87815 | 0.275187 | 10.4589 | 1.33E-25 | 1.37E-22 |
| BAALC | 508.001 | 2.08162 | 0.344216 | 6.04744 | 1.47E-09 | 1.16E-07 |
| DES | 109.845 | 1.97305 | 0.379097 | 5.2046 | 1.94E-07 | 9.27E-06 |
| ARHGAP28 | 170.851 | 1.89048 | 0.304053 | 6.2176 | 5.05E-10 | 4.54E-08 |
| TMEM119 | 2000.18 | 1.7824 | 0.385105 | 4.62836 | 3.69E-06 | 0.000115083 |
| WISP1 | 1513.13 | 1.76239 | 0.290793 | 6.06063 | 1.36E-09 | 1.07E-07 |
| SYTL2 | 595.359 | 1.56253 | 0.229966 | 6.79462 | 1.09E-11 | 1.45E-09 |
| SLC1A3 | 189.588 | 1.53515 | 0.353692 | 4.34036 | 1.42E-05 | 0.000358588 |
| CRISPLD1 | 85.1122 | 1.53249 | 0.360657 | 4.24915 | 2.15E-05 | 0.000509689 |
| BACH2 | 70.2948 | 1.51496 | 0.356655 | 4.24768 | 2.16E-05 | 0.000510714 |
| **RUNX2** | 652.978 | 1.50187 | 0.217413 | 6.90794 | 4.92E-12 | 7.22E-10 |
| SHF | 33.8572 | 1.46041 | 0.383853 | 3.8046 | 0.000142031 | 0.0024871 |
| TNFRSF19 | 589.791 | 1.42522 | 0.210637 | 6.76622 | 1.32E-11 | 1.73E-09 |
| LSP1 | 124.033 | 1.3613 | 0.366004 | 3.71936 | 0.000199728 | 0.00328558 |
| C7orf31 | 31.217 | 1.28882 | 0.378487 | 3.40519 | 0.000661189 | 0.0085924 |
| TNC | 21203.3 | 1.1748 | 0.228728 | 5.13622 | 2.80E-07 | 1.27E-05 |
| TGFB3 | 311.25 | 1.12405 | 0.261592 | 4.29697 | 1.73E-05 | 0.00042451 |
| CD59 | 41119.2 | -1.03487 | 0.204063 | -5.07132 | 3.95E-07 | 1.68E-05 |
| RNF144B | 129.381 | -1.23448 | 0.313597 | -3.93653 | 8.27E-05 | 0.00158657 |
| NTN4 | 8416.76 | -1.23814 | 0.267545 | -4.62778 | 3.70E-06 | 0.000115154 |
| FAM167A | 344.698 | -1.2398 | 0.24855 | -4.98814 | 6.10E-07 | 2.44E-05 |
| ARHGAP29 | 5019.98 | -1.27064 | 0.26399 | -4.81322 | 1.49E-06 | 5.34E-05 |
| HECW2 | 755.074 | -1.34131 | 0.21951 | -6.1105 | 9.93E-10 | 8.26E-08 |
| MYOZ2 | 35.6334 | -1.36412 | 0.385145 | -3.54184 | 0.000397349 | 0.00573665 |
| HDAC9 | 196.788 | -1.38978 | 0.26451 | -5.25417 | 1.49E-07 | 7.28E-06 |
| IL17D | 160.017 | -1.43611 | 0.342481 | -4.19325 | 2.75E-05 | 0.000625301 |
| TEK | 1004.77 | -1.43726 | 0.164397 | -8.74264 | 2.28E-18 | 9.11E-16 |
| SFTA1P | 52.591 | -1.44078 | 0.385001 | -3.74228 | 0.00018236 | 0.00304159 |
| PERP | 3655.17 | -1.46707 | 0.212762 | -6.89535 | 5.37E-12 | 7.77E-10 |
| LRRN3 | 126.364 | -1.475 | 0.324836 | -4.54077 | 5.60E-06 | 0.000162329 |
| SGIP1 | 96.9042 | -1.5039 | 0.383017 | -3.92647 | 8.62E-05 | 0.00163045 |
| SDPR | 92.9302 | -1.54665 | 0.361729 | -4.27572 | 1.91E-05 | 0.00046089 |
| ADRB2 | 58.1689 | -1.5683 | 0.379305 | -4.13466 | 3.55E-05 | 0.000772935 |
| RGS7 | 52.7216 | -1.5684 | 0.375847 | -4.17298 | 3.01E-05 | 0.00067407 |
| SMURF2 | 8577.41 | -1.57164 | 0.209358 | -7.50695 | 6.05E-14 | 1.24E-11 |
| EDN1 | 744.547 | -1.57221 | 0.239329 | -6.56921 | 5.06E-11 | 5.87E-09 |
| CD36 | 84.5038 | -1.59805 | 0.369687 | -4.32272 | 1.54E-05 | 0.00038514 |
| HIST1H2BC | 78.1792 | -1.6057 | 0.377277 | -4.25602 | 2.08E-05 | 0.000495096 |
| FGD4 | 435.407 | -1.64201 | 0.353052 | -4.65089 | 3.31E-06 | 0.000105952 |
| SH3TC2 | 89.5097 | -1.65136 | 0.339273 | -4.86735 | 1.13E-06 | 4.21E-05 |
| ALDH1A3 | 4967.92 | -1.66157 | 0.250644 | -6.6292 | 3.38E-11 | 4.05E-09 |
| NMNAT2 | 646.204 | -1.77977 | 0.233826 | -7.6115 | 2.71E-14 | 5.91E-12 |
| TLR4 | 2152.78 | -1.86538 | 0.229706 | -8.12075 | 4.63E-16 | 1.33E-13 |
| SCN9A | 1853.36 | -1.89703 | 0.268985 | -7.05254 | 1.76E-12 | 2.76E-10 |
| HIST1H2BK | 1615.59 | -2.03662 | 0.256646 | -7.93555 | 2.10E-15 | 5.29E-13 |
| NPAS1 | 378.928 | -2.05877 | 0.233902 | -8.80185 | 1.35E-18 | 5.87E-16 |
| CCND1 | 54719.9 | -2.0907 | 0.2371 | -8.81778 | 1.17E-18 | 5.25E-16 |
| PCDH10 | 599.913 | -2.82787 | 0.269676 | -10.4862 | 1.00E-25 | 1.11E-22 |
| **PLAT** | 44638.1 | -3.0236 | 0.297923 | -10.1489 | 3.35E-24 | 2.68E-21 |
