# Supplementary figures and images for: Single cell sequencing reveals gene expression signatures associated with bone marrow stromal cell subpopulations and time in culture
Source: J Transl Med. 2019 Jan 11;17:23. doi: 10.1186/s12967-018-1766-2 (PMC6330466; doi:10.1186/s12967-018-1766-2)

# Additional File 3: Figure S1

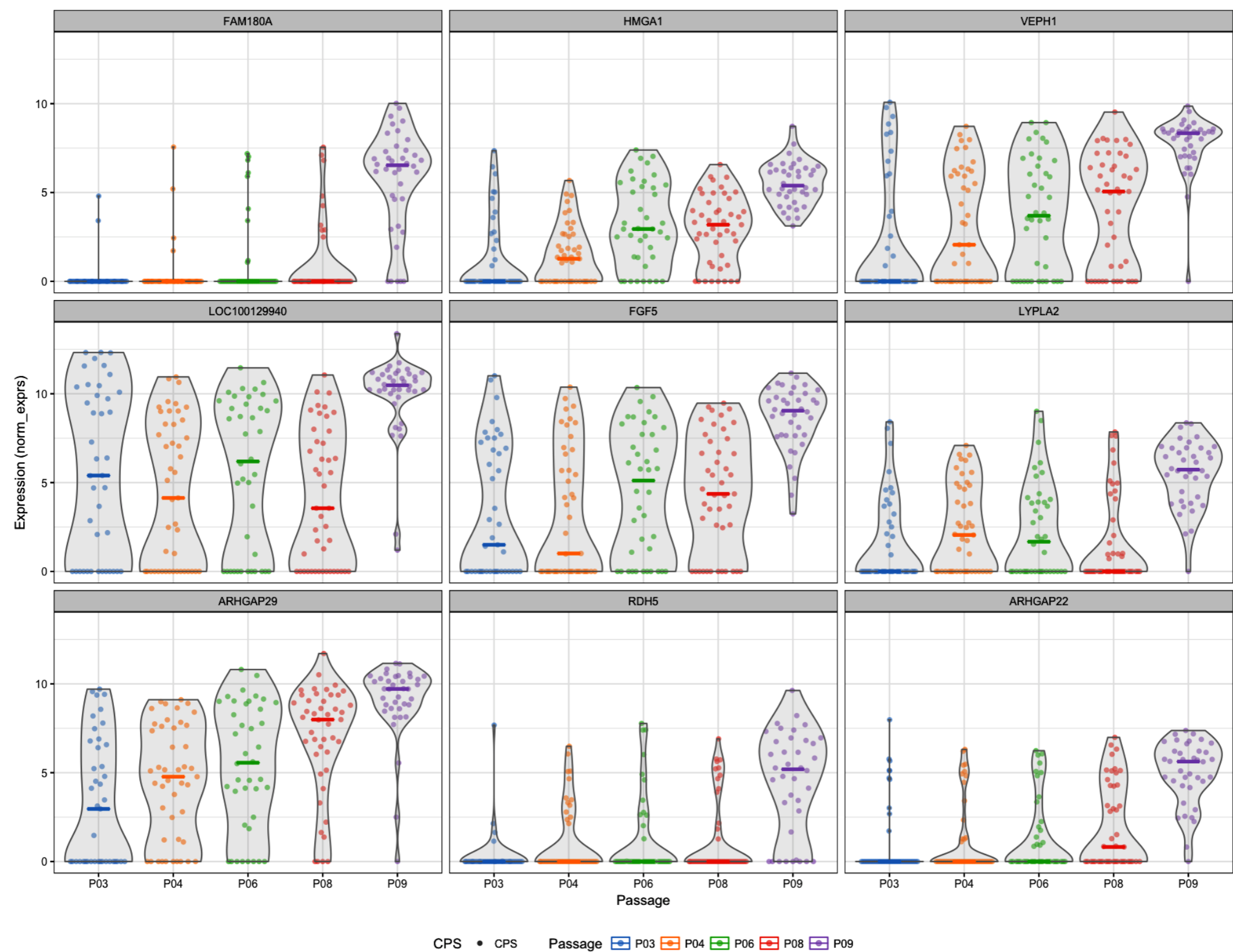

Supplement: Supplementary file 3 — Additional file 3: Figure S1. Gene expressions in single cells. [file 12967_2018_1766_MOESM3_ESM.pdf]
